# On the relationship between glucose absorption and glucose‐stimulated secretion of GLP‐1, neurotensin, and PYY from different intestinal segments in the rat

**DOI:** 10.14814/phy2.13507

**Published:** 2017-12-04

**Authors:** Rune E. Kuhre, Charlotte B. Christiansen, Monika Y. Saltiel, Nicolai J. Wewer Albrechtsen, Jens J. Holst

**Affiliations:** ^1^ Department of Biomedical Sciences Faculty of Health and Medical Sciences University of Copenhagen Copenhagen Denmark; ^2^ NNF Center for Basic Metabolic Research Faculty of Health and Medical Sciences University of Copenhagen Copenhagen Denmark

**Keywords:** Glucagon‐like peptide‐1, glucose absorption, isolated perfused intestine, neurotensin, peptide YY

## Abstract

Ingested glucose powerfully stimulates the secretion of appetite‐ and metabolism‐regulating peptide hormones from the gut – including glucagon‐like peptide‐1 (GLP‐1), neurotensin (NT), and polypeptide YY (PYY). However, the regional origin of these secretions after glucose stimulation is not well characterized, and it remains uncertain how their secretion is related to glucose absorption. We isolated and perfused either the upper (USI) or the lower (LSI) small intestine or the colon from rats and investigated concomitant glucose absorption and secretory profiles of GLP‐1, NT, and PYY. In the USI and LSI luminal glucose (20%, w/v) increased GLP‐1 and NT secretion five to eightfold compared to basal secretion. Compared to the USI, basal and stimulated GLP‐1 secretion from the colon was 8–10 times lower and no NT secretion was detected. Luminal glucose stimulated secretion of PYY four to fivefold from the LSI and from the USI and colon, but the responses in the USI and colon were 5‐ to 15‐fold lower than in the LSI. Glucose was absorbed to a comparable extent in the USI and LSI by mechanisms that partly depended on both SGLT1 and GLUT2 activity, whereas the absorption in the colon was 80–90% lower. The absorption rates were, however, similar when adjusted for segmental length. Glucose absorption rates and NT, PYY and in particular GLP‐1 secretion were strongly correlated (*P* < 0.05). Our results indicate that the rate of secretion of GLP‐1, NT, and PYY in response to glucose, regardless of the involved molecular machinery, is predominantly regulated by the rate of glucose absorption.

## Introduction

The gut peptide hormones, glucagon‐like peptide‐1 (GLP‐1), and peptide YY (PYY) play an important role in the regulation of appetite and food intake (Baggio and Drucker 2007; Steinert et al. 2017; Tan et al. 2017), and neurotensin secreted from intestinal N‐cells may also inhibit appetite (Levine et al. 1983). GLP‐1, in addition, potentiates glucose‐stimulated insulin secretion and thus contributes to the incretin effect (Kreymann et al. 1987). Over the last two decades, substantial efforts have been made to uncover the molecular mechanisms underlying nutrient‐stimulated secretion of these peptide hormones (Gribble and Reimann 2016; Husted et al. 2017), but the regional anatomical origin of their secretion and its relation to macronutrient absorption remains unclear. Based on immunohistochemistry, it appears that in humans NT, GLP‐1 and in particular PYY‐positive cells are more abundant in the distal small intestine and in the colon (PYY and GLP‐1) (Varndell et al. 1985; Evers et al. 1993; Theodorakis et al. 2006), and mucosal concentrations of extractable peptides show a similar pattern in mice and rats (Larsson et al. 1975; Larsson and Rehfeld 1978; Evers et al. 1993; Svendsen et al. 2015; Wewer Albrechtsen et al. 2016). However, there is no simple relationship between tissue concentration or number of “specific” enteroendocrine cells and actual hormone secretion (Svendsen et al. 2015; Gribble and Reimann 2016; Kuhre et al. 2016). For instance, the high concentration of these peptides in the colon raises the question whether a similarly large secretion can be elicited from this part of the gut. However, the secretion in response to a given nutrient depends on the expression of the molecular machinery that is activated by nutrients, and this may vary between cells from different parts of the intestine. Recent studies in people with gastric bypass surgery indicate that the exaggerated increase in GLP‐1, NT, and PYY secretion after oral glucose intake is related to the rate of glucose *absorption* rather than the *presence* of glucose in the gut (Jørgensen et al. 2012; Jacobsen et al. 2013), and this assumption is supported by in vitro experiments (Parker et al. 2012; Kuhre et al. 2014a, 2014b). Nevertheless, this apparently important relationship has not, to our knowledge, been studied directly in a quantitative manner. Therefore, the purpose of this study was to examine the potential relationship between glucose absorption and glucose‐stimulated GLP‐1, NT, and PYY secretion from the different intestinal sections. For this, we used isolated perfused segments from the rat gut: the upper and lower half of the small intestine and the colon. In our perfusion models, all the important physiological parameters such as cell polarization, contact with normal neighbor cells, nutrition, and respiration and most importantly for studies of transmucosal transport: perfusion flow (and therefore convective drag of absorbed nutrients) are preserved (Svendsen and Holst 2016), allowing studies of the full dynamics of absorption and secretion.

## Material and Methods

### Ethical considerations

Studies were conducted with permission from the Danish Animal Experiments Inspectorate (2013‐15‐2934‐00833) and the local ethical committee and were conducted in accordance with the EU Directive 2010/63/EU and guidelines of Danish legislation governing animal experimentation (1987) and the NIH (publication number 85–23).

### Isolated perfused rat intestine

Male Wistar rats (~250 g) were obtained from Janvier (Saint Berthevin Cedex, France) and housed two per cage with ad libitum access to drinking water and chow, following a 12 h light/dark cycle. At the day of experiment, nonfasted rats were anesthetized with a subcutaneous injection of Hypnorm/midazolam (0.079 mg fentanyl citrate + 2.5 mg fluanisone + 1.25 mg midazolam/mL: 0.3 mL/100 g bodyweight). Experiments were conducted between 09.00 and 10.30 and between 13.00 and 14.00 (equal number of rats at the two times) meaning that the rats, since they had been exposed to light from 07.00 am, had been “semi‐fasted” for either 2–3 or 6–7 h prior to the experiment. This difference in fasting time did not affect glucose absorption or hormone secretion (data not shown). Rats were placed on a heated operating table (37°C), the abdominal cavity was opened, and all parts of the intestine not to be included for perfusion were removed after ligation of the supplying vasculature. The remaining intestine (either the upper or lower half of the small intestine (USI or LSI) or the colon) was left in situ in the animal – still connected to its vasculature. The USI included the segment from the duodenum until approximately 50 cm from cecum, whereas the LSI included the lower approximately 50 cm of the small intestine including the terminal ileum boarding up to the ileo‐cecal valve. A tube was placed in the lumen and the luminal contents were emptied by gently flushing with isotonic saline (room temperature). Thereafter, a steady flow of saline was applied until the end of experiment (USI and LSI = 0.5 mL/min, colon = 0.05 mL/min), except for the stimulation periods were glucose was administered as described below. The retained intestine was vascularly perfused (USI and LSI: 7.5 mL/min, colon 3 mL/min) via a catheter inserted into the upper mesenteric artery (a. mesenterica sup.) in case of the small intestine or the aorta for the colon and perfused with perfusion buffer using a UP100 Universal Perfusion System from Hugo Sachs (Harvard Apparatus, March Hugstetten, Germany) heating the buffer to 37°C. For the colon preparation, the kidneys were tied off, the spleen, stomach, and small intestine were removed and the celiac artery (partly perfusing the pancreas) were ligated to ensure that only the colon was perfused. Perfusion buffer was a Krebs‐Ringer bicarbonate buffer supplemented with 0.1%(w/v) BSA (Fraction V; cat. no. 1.12018.0500, Merck, Ballerup, Denmark), 5% (w/v) dextran T70 (to balance osmolarity, Pharmacosmos, Holbaek, Denmark), 2% (v/v) amino acid mixture (Vamin, Cat. no. 11,338, Fresenius Kabi, Uppsala, Sweden), 3.5 mmol/L glucose, 10 *μ*mol/L 3‐isobutyl‐1‐methylxanthine (Cat. no.5879, Sigma Aldrich), and 5 mmol/L pyruvate, fumarate and glutamate. The buffer was oxygenated by gassing with 95% O_2_/5% CO_2_ and pH adjusted to 7.4–7.5. Venous effluent samples were collected each minute from a catheter inserted into the vena portae, and samples were immediately put on ice. As soon as perfusion flow was established, the rat was euthanized by perforation of the diaphragm, and the selected gut segment was perfused for approximately 30 min before the experiment was started. For the present experiments, the stimulant was intraluminal glucose (20% w/v) dissolved in isotonic saline (room temperature) delivered with an initial bolus to replace the saline in the intestine (USI and LSI: 2.5 mL/min for three minutes, colon: 0.25 mL/min for five minutes) followed by a slower infusion rate for the remainder of the stimulation period (USI and LSI: 0.5 mL/min, colon: 0.15 mL/min). At the end of the stimulation period, the lumen was flushed with isotonic saline (room temperature) at same rates as described above. In separate experiments the entire small intestine (103.7 ± 5.4 cm, except ~3 cm of the most proximal part) was perfused as described above (7.5 mL/min) and glucose absorption was investigated in response to administration of 100 mmol/L glucose ± 10 mmol/L phloridzin (SGLT1 inhibitor) and 1 mmol/L phloretin (GLUT2 inhibitor). Because of the longer length of the retained intestine, the luminal administrations was in this case given as an initial bolus of 2.5 mL/min for 5 min followed by 10 min at 0.5 mL/min. Perfusion pressure and effluent flow rate were closely monitored throughout the experiment and used as an indication of the wellbeing of the perfused intestinal segment. Both parameters remained stable during all reported experiments. Therefore, the measured peptide concentrations in the venous effluent reflect the actual secretory output (fmol/min = concentration (fmol/L) × flow (L/min)). Length of retained intestine varied little within groups (coefficient of variations: USI = 10.2%, LSI = 6.89%, colon = 9.41%) but differed between the USI or LSI and the colon (mean ± SEM: USI = 45.88 ± 2.10 cm, LSI = 48.00 ± 1.65 cm, colon = 9.81 ± 0.92 cm, *P*_USI or LSI_ vs. colon < 0.01) but not between USI and LSI (*P* = 0.11). Samples were stored at −20°C until analysis. The experimental set up has been described in more detail elsewhere (Kuhre et al. 2014a,b).

### Expression analysis

Rats were anesthetized as described above and tissue was harvested, thoroughly rinsed in 0.9% NaCl (room temperature) and transferred to RNA‐later and stored at 4°C. Total RNA was extracted from homogenized rat intestine tissue (full thickness tissue) using TRIzol^®^ (Invitrogen). RNA (1 *μ*g) was treated with DNase I and reverse‐transcribed using the High‐Capacity cDNA RT (reverse transcription) kit from Applied Biosystems. qPCR was performed using SYBR Green PCR Mastermix from Applied Biosystems and a program of: 2 min at 50°C and 10 min at 95°C, followed by 40 cycles of 15 sec at 95°C and 60 sec at 60°C. Sodium‐glucose cotransporter 1 (SGLT1 aka. solute carrier family 5 member 1 (Slc5a1) mRNA was quantified by use of following primers: Forward: CAAGATCCGGAAGGGTGCAT: backward: TGTAGTCAAAGAGCTGCCCG. rPO (ribosomal phosphoprotein) was used as a reference gene, and qPCR data were analyzed using the 2(−*∆∆ct*) method. Samples were analyzed in triplicates.

### Biochemical measurements

Glucose concentration in perfusion effluent was measured by a handheld glucometer, based on the glucose‐oxidation method (Accu‐chek Compact plus device; Roche, Mannheim, Germany). Coefficient of variation was less than 20% in the range 0.1–1 mmol/L. GLP‐1 concentration in venous effluent was measured by in‐house RIA (assay code: 89‐390) based on a so‐called “terminal‐wrapping” antibody recognizing the amidated c‐terminal part of the molecule, thus measuring *total* GLP‐1 (1–36NH_2_, 7–36NH_2_, and 9–36NH_2_), but not glycine extended isoforms of GLP‐1 x ‐37(Orskov et al. 1994). The assay has a detection limit of 1 pmol/L with an intraassay coefficient of variation of 6%.The choice of targeting amidated isoforms of the molecule was based on previous studies from our group showing that the rat intestine predominantly stores amidated GLP‐1 (Kuhre et al. 2014a). Similarly, *total* NT and *total* PYY were measured using in‐house RIA assays, employing C‐terminally targeting antibodies for both peptides, thus measuring both intact (NT 1–13 and PYY 1–36) and cleaved isoforms ((Kuhre et al. 2015); (Torang et al. 2016)). For the NT assay, the detection limit is 2 pmol/L with an intraassay coefficient of variation of 9% and the PYY assay had comparable characteristics.

### Data presentation and statistics

Data are presented as means ± SEM for perfusion data. Statistical analysis was applied to test if hormone outputs differed between baseline and during glucose stimulation and if the secretory output during glucose instillation differed between the respective intestinal segments. Glucose absorption was calculated by subtracting the arterial glucose supply (3.5 mmol/L × flow, corresponding to a delivery of 26.25 *μ*mol/min to the USI and LSI and 10.5 *μ*mol/min to the colon) from the venous glucose outputs. Response outputs (*n *=* *7–9) were calculated over a period from start of glucose administration until the end of stimulation (indicated in the graphs), and baseline outputs were calculated for the same number of minutes as the stimulation period. Correlations between glucose absorption (*μ*mol) and hormone outputs (fmol) were calculated by linear regression analysis. Total glucose absorption at baseline (*μ*mol, 10 min preceding the luminal glucose administration) and during luminal glucose administration (*μ*mol, also a 10 min period) was plotted against total hormone output in the same periods. 95% confidence intervals are depicted in the respective graphs. *Expression data* are expressed relative to the concentration in the first tissue sample (1 cm from pylorus). Within group significance (*n* = 7–9) was assessed by paired *t*‐test (perfusions) or by one‐way‐ANOVA for repeated measurements between group comparison for the perfusion data and expression analysis data (*n* = 3)) followed by Tukey's post hoc test. Between group significance (perfusion data, baseline subtracted secretory outputs in response to glucose stimulation, *n* = 7–9) was evaluated by one‐way ANOVA followed by Tukey's post hoc test. For all tests, *P* < 0.05 was considered significant. Calculations were made using GraphPad Prism 6 software (La Jolla, CA) and graphs were constructed in GraphPad and edited in Adobe Illustrator.

## Results

### Glucose absorption

#### Isolated rat intestine perfusions

**Figure 1:**
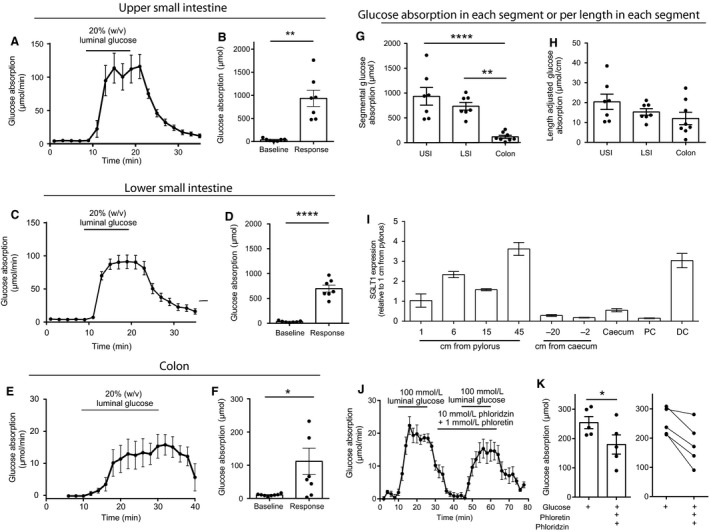
Glucose absorption from the rat intestine. Data are shown as means ± SEM. (A, C, E) Glucose absorption following glucose administration in the upper (A) or lower half (C) of the small intestine or colon (E) as indicated. (B, D, F) Corresponding total glucose absorption during glucose administration. (G) Segmental total glucose absorption during glucose administration (subtracted baseline). (H) Length adjusted glucose absorption during luminal glucose administration (also subtracted baseline). (I) Expression of sodium‐glucose cotransporter‐1 (SGLT1) along the rat intestine. (J) Glucose absorption in the absence or presence of luminal inhibition of SGLT1 and GLUT2 activity. (K) Corresponding total glucose absorption during glucose administration. Individual observations are indicated with dots. *P < 0.05, **P < 0.01, ****P < 0.0001. *n*: A–D = 7, E, F = 7, I = 3, J, K = 5.

Intraluminal glucose administration in the USI rapidly increased glucose absorption (baseline = 3.75 ± 1.1 *μ*mol/min vs. stimulated = 93.5 ± 18 *μ*mol/min, *P* < 0.01, *n* = 7, Fig. 1A and B). A similar increase in glucose absorption was observed from the LSI after luminal glucose (baseline = 3.21 ± 0.6 *μ*mol/min vs. stimulation = 70.0 ± 6.9 *μ*mol/min, Fig. 1C and D), whereas glucose output from the colon increased twofold in 6/8 experiments (Fig. 1E and F) but was on average six to ninefold lower than the total absorption in the USI and LSI over a 10‐min period (USI = 935 ± 177 *μ*mol, LSI = 735.1 ± 78 *μ*mol, colon = 107.7 ± 37 *μ*mol, *n *=* *6–7, *P*_USI vs. LSI_ = 0.46, P_USI vs. colon_ < 0.001, *P*_LSI vs. colon_ < 0.001, *n* = 6–8, Fig. 1G). However, the difference between the USI and LSI compared to the colon mainly resulted from the difference in segmental lengths, as the incremental glucose absorption (during response) per cm was not significantly different: USI = 20.41 ± 1.62 *μ*mol/cm, LSI = 15.31 ± 1.62 *μ*mol/cm, colon: 12.01 ± 3.1 *μ*mol/cm (*P* > 0.05 for all groups, Fig. 1I).

#### Tissue expression of sodium‐glucose cotransporter 1 (SGLT1)

SGLT1 expression was mainly restricted to the upper 2/3 of the small intestine with low expression in the terminal part of the small intestine, the cecum and proximal part of the colon. In the distal part of the colon, SGLT1 was, however, expressed at levels comparable to those found 45 cm from pylorus (the site of highest expression in the small intestine) (Fig. 1I¸ *n* = 3).

#### SGLT1‐ and GLUT2‐mediated glucose absorption

Having established that the small intestine is the major site of glucose absorption, we next investigated the role of the glucose transporters SGLT1 and GLUT2 for glucose absorption in this intestinal segment. Luminal glucose (100 mmol/L) was rapidly absorbed, but the absorption was reduced by approximately 30% upon SGLT1 and GLUT2 inhibition with phloridzin and phloretin, respectively (absorption in response to: glucose = 254.2 ± 20.7 *μ*mol, glucose + phloridzin + phloretin = 179.6 ± 32.6 *μ*mol, Fig. 1J and K, *n* = 5, *P* = 0.012). The attenuated glucose absorption was not related to a generally reduced glucose absorption at the second time of glucose administration as the absorption at the second time of glucose administration (20% w/v) without inhibitors was identical to that of the first administration (glucose absorption: first stimulation = 508.0 ± 101 *μ*mol vs. second stimulation = 487.8 ± 110.9, *n* = 6, *P* = 0.70, data not shown).

### Glucose‐stimulated GLP‐1, NT, and PYY secretion from the upper and lower small intestine and the colon and the relationship with glucose absorption

#### GLP‐1

**Figure 2:**
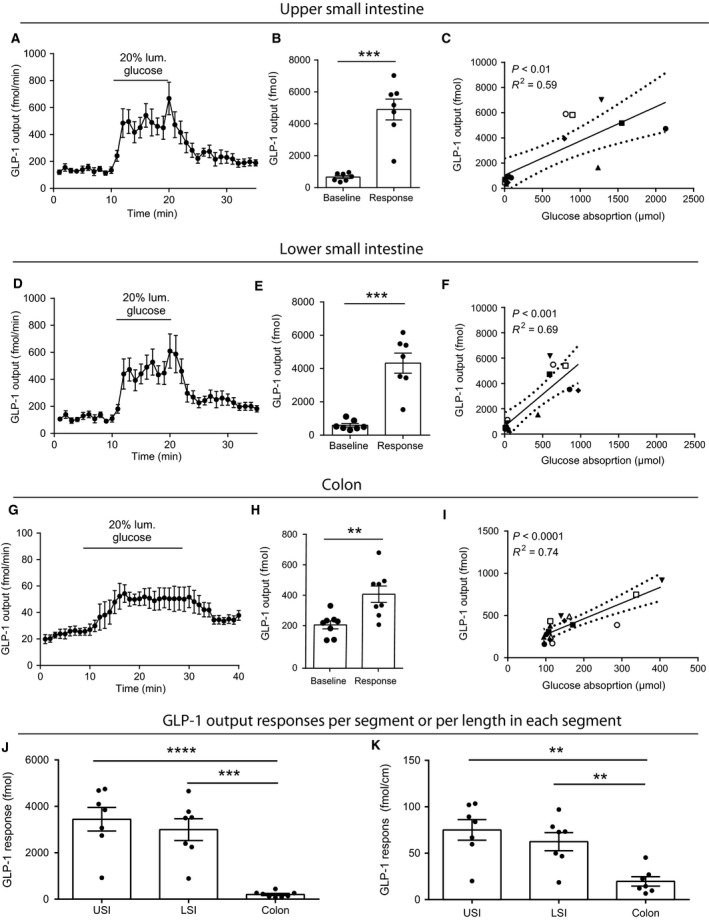
Glucose‐stimulated GLP‐1 (total) secretion from isolated perfused rat small intestine. Data are shown as means ± SEM. (A–C) Upper half of small intestine. (D–F**)** Lower half of the small intestine. (J) Glucose‐stimulated GLP‐1 responses from upper and lower small intestine and colon (baseline subtracted), (K) Length adjusted glucose‐stimulated GLP‐1 responses in the upper and lower small intestine and colon (also baseline subtracted). Individual observations are indicated with dots. Correlation analysis between glucose absorption (*μ*mol) and GLP‐1 output (fmol) is shown for the respective anatomical segments in C, F, and I. Staged lines indicate 95% confidence intervals. Symbols indicate different animals (two symbols/animal). **P < 0.01, ***P < 0.001, ****P < 0.001, *n*: A–C = 7, D–F = 7, G–I = 8, J and K = 7–8).

Glucose induced robust and similar GLP‐1 responses from the USI and the LSI, with total secretion increasing approximately eightfold compared to baseline (GLP‐1 output during a similar time span immediately preceding the stimulation (USI: baseline output = 0.67 ± 0.90 pmol vs. stimulated output = 4.90 ± 0.65 pmol, LSI: baseline output 0.66 ± 0.11 pmol vs. response = 4.33 ± 0.60 pmol, *P* < 0.001 compared to respective baselines, Fig. 2A–F). Basal GLP‐1 secretion from the *colon* was approximately fivefold lower and glucose only elicited a modest, but significant, increase in GLP‐1 secretion (output baseline = 0.22 ± 0.03 pmol vs. response = 0.41 ± 0.05 pmol, *P* < 0.01, *n* = 8, Fig. 2G–I). Overall, the glucose‐stimulated GLP‐1 response was approximately 10‐fold greater from the USI and LSI compared to the colon but did not differ significantly between USI and LSI (baseline subtracted outputs during glucose infusion: USI = 3.4 ± 0.5 pmol, LSI = 3.0 ± 0.5 pmol, colon 0.2 ± 0.04 pmol, *P*_USI vs. LSI_ = 0.70, *P*_USI or LSI vs. colon_ < 0.001, *n* = 6–8, Fig. 1J). In the USI, LSI, and colon, GLP‐1 secretion correlated positively with glucose absorption rate (USI: *R* ^2^ = 0.59, LSI: *R* ^2^ = 0.69, colon: *R* ^2^ = 0.74, all *P* < 0.01, Fig. 2C, F, and I). Adjusted for segmental length, the secretory GLP‐1 response to glucose from the USI was comparable to that from the LSI, and both were 3–4 times larger than the secretion from colon (baseline subtracted secretory output during glucose infusion: USI = 75 ± 11 fmol/cm, LSI = 62 ± 10 fmol/cm, colon = 20 ± 5 fmol/cm, *P*_USI vs. LSI_ = 0.59, *P*_USI or LSI vs. colon_ < 0.01, Fig. 2K). In all cases, the regression line passed close to 0.0, suggesting that there was little secretion without glucose absorption.

#### NT

**Figure 3:**
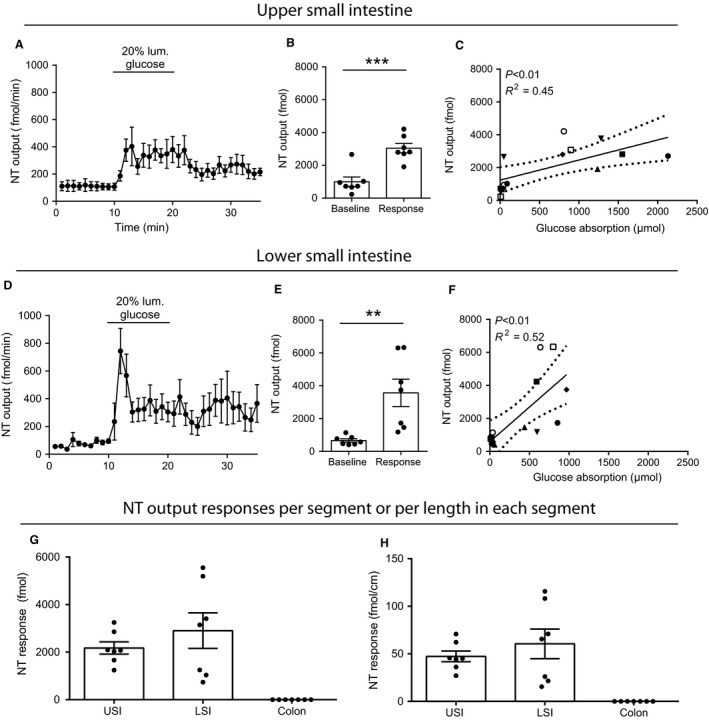
Glucose‐stimulated NT (total) secretion from isolated perfused rat small intestine. Data are shown as means ± SEM. (A–C) Upper half of small intestine. (D–F) Lower half of the small intestine, (G) Glucose‐stimulated NT responses from upper and lower small intestine and colon (baseline subtracted), (H) Length adjusted glucose‐stimulated NT secretion from the upper and lower small intestine and colon (also baseline subtracted). Individual observations are indicated with dots. Correlation analysis between glucose absorption (*μ*mol) and NT output (fmol) is shown for the upper and lower half of the small intestine in C and F. Staged lines indicate 95% confidence intervals. Symbols indicate to different animals (two symbols/animal). No immunoreactive NT was detected in colon perfusates, neither at baseline nor in response to glucose (data not shown). **P <* *0.01, ***P <* *0.001, *n*: A–C = 7, D–F = 8, G and H = 7–8

Glucose generated similar NT responses from the USI and LSI, resulting in about fivefold increase in total NT output during glucose infusion (response) compared to the basal secretion over a similar time interval immediately preceding stimulation (USI: 1.0 ± 0.3 pmol vs. 3.0 ± 0.3 pmol, LSI: 0.7 ± 0.1 pmol vs. 3.6 ± 0.8 pmol, both *P* < 0.01 compared to respective baselines, incremental outputs: USI = 2.2 ± 0.3 pmol vs. 2.9 ± 0.7 pmol, *P* = 0.22, *n* = 7, Fig. 3A, B, D, E, and G). In contrast, NT was not secreted from the colon in detectable amounts (lower limit of quantification ~ 1 pM, data not shown). NT secretion and glucose absorption correlated positively but to a lower extent than the case for GLP‐1 secretion (USI: *R* ^2^ = 0.45, *P* < 0.01, LSI: *R* ^2^ = 0.52, *P* < 0.01, Fig. 3C and F). Probably the difference is driven by that NT plateaued already at small increases in glucose absorption (output) (Fig. 3C and F). Glucose‐stimulated NT secretion did not differ between the USI and LSI when adjusted for segmental length (baseline‐subtracted outputs (fmol/cm): USI = 47.35 ± 5.61, LSI = 60.50 ± 15.6, *P* = 0.44, Fig. 3H).

#### PYY

**Figure 4:**
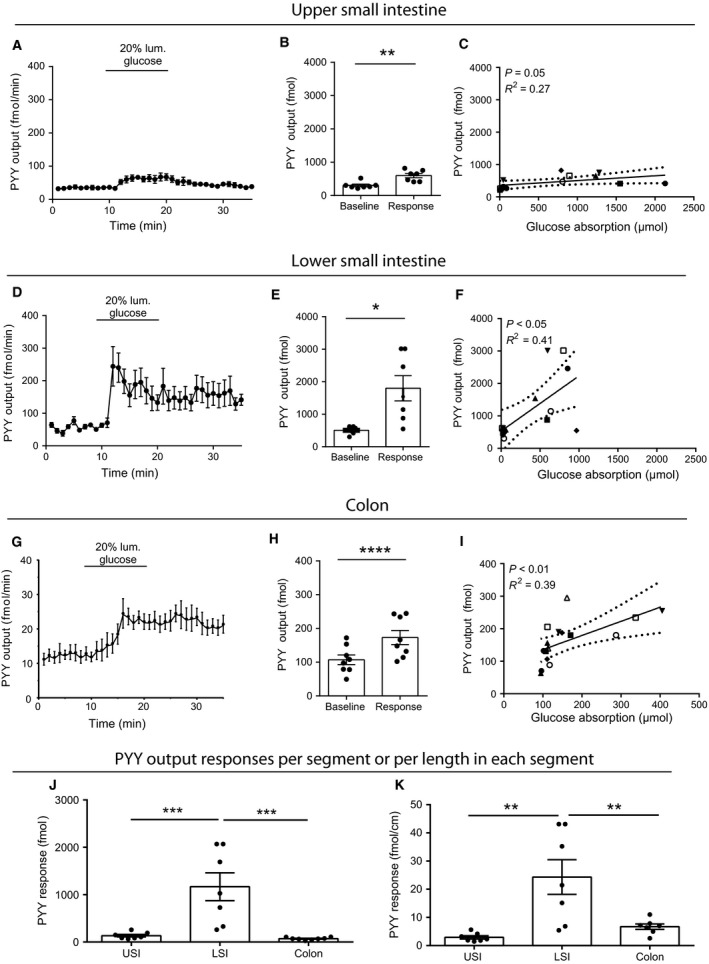
Glucose‐stimulated PYY(total) secretion from isolated perfused rat small intestine. Data are shown as means ± SEM. (A–C) Upper half of small intestine, (D–F) Lower half of the small intestine, (G–I) colon. (J) Glucose‐stimulated PYY responses from upper and lower small intestine and colon (baseline subtracted). (K) Length adjusted glucose‐stimulated PYY secretion from the upper and lower small intestine and colon (also baseline subtracted). Individual observations are indicated with dots. Correlation analysis between glucose absorption rate (*μ*mol) and PYY output (fmol) is shown for the respective anatomical segments in C, F, and I. Staged lines indicate 95% confidence intervals. Symbols indicate different animals *P < 0.05, **P < 0.01, ****P < 0.0001. *n*: A–C = 7, D–F = 7, G–I = 8, J and K = 7–8).

Glucose elicited a small but significant secretory PYY response from the USI (basal secretion = 0.3 ± 0.04 pmol vs. 0.6 ± 0.06 pmol, *P* < 0.05, Fig. 4A and B) and from the colon (basal secretion = 0.1 ± 0.01 pmol vs. 0.2 ± 0.02 pmol, *P* < 0.001, Fig. 4G and H), but PYY was predominately secreted from the LSI (Fig. 4D and E), where glucose administration resulted in a secretory response that was 10‐ to 15‐fold higher than those from the USI and colon (outputs (pmol): USI = 1.3 ± 0.03, LSI = 1.2 ± 0.3, colon = 0.07 ± 0.01, *P*_LSI vs. USI or colon_ < 0.001, *n* = 7–8, Fig. 4J). The association between PYY secretion and glucose absorption was: USI: *R* ^2^ = 0.27, *P* = 0.05, LSI =* R* ^2^ = 0.41, *P* = 0.05, colon: *R* ^2^ = 0.39, *P* < 0.01 in all cases, Fig. 4C, F, and I). Taking the difference in segmental length into account, the secretory potential per cm was 3–8 times larger in the LSI than in the USI and colon, respectively (length adjusted incremental outputs (fmol/cm): USI = 2.93 ± 0.6, LSI = 24.3 ± 6.1, colon = 6.7 ± 1.0, *P*_LSI vs. USI or colon_ < 0.01 Fig. 4K).

## Discussion

Several studies have addressed the molecular mechanisms underlying the secretion of the appetite‐ and metabolism‐regulating hormones, GLP‐1, NT, and PYY during the last decade, as recently reviewed (Gribble and Reimann 2016; Husted et al. 2017), but less attention has been devoted to analysis of the segments of the gut from where these hormones are secreted in response to different secretagouges (e.g., amino acids, bile acids, glucose, short‐ and long‐chain fatty acids, and proteins). Clearly, the expression levels of different molecular sensors in intestinal epithelium of mice and humans vary along the intestine (Symonds et al. 2015) and this may result in different regional responses of GLP‐1, NT, and PYY to different stimuli. In particular, the colon has only to a limited extent been studied for its endocrine secretion, albeit traditional analysis (immunohistochemistry) of human and rat intestinal sections has demonstrated that the highest density of the GLP‐1 producing L cells is found here (Larsson et al. 1975; Larsson and Rehfeld 1978; Varndell et al. 1985; Evers et al. 1993; Theodorakis et al. 2006 Svendsen et al. 2015; Wewer Albrechtsen et al. 2016). However, conventional analysis does not take into account the differences in surface area and length of the different regions of the intestine; if this is done, one may find in humans that although sparse in density, the total L‐cell numbers are higher in the upper jejunum and ileum than in the large intestine (Nauck 1998), and experimental (unbiased) stereology data from rats have shown that L cells are distributed rather evenly throughout the jejunum, ileum, and colon (Hansen et al. 2013).

In humans, glucose ingestion elevates plasma NT, GLP‐1, and PYY levels (Jacobsen et al. 2012), and glucose appears in healthy subjects to be the most effective secretagogue for GLP‐1 (Jensen et al. 2016). Studies from several independent groups have suggested that glucose‐stimulated GLP‐1 and NT secretion relies on L‐/N‐cell mediated uptake of glucose through SGLT1, which gives rise to depolarization of the N/L cell, opening of voltage‐gated calcium channels and influx of extracellular calcium (Gribble et al. 2003; Parker et al. 2009; Gorboulev et al. 2012; Mace et al. 2012; Kuhre et al. 2014b, 2015; Röder et al. 2014; Gribble 2015) although a contribution from metabolism of glucose and GLUT2 depending mechanisms may also play a role (Mace et al. 2012; Kuhre et al. 2014b). In addition, little is known about the relative expression of SGLT1 in GLP‐1, NT, and PYY‐positive cells in the upper and lower small intestine and in the colon, and the ability of the endocrine cells in the different parts of the intestine to respond to and/or transport glucose is also unknown. Our results show that glucose is absorbed by and stimulated GLP‐1 secretion from the USI and LSI to a comparable extent, whereas both absorption and secretion from the colon was 10‐ to 15‐fold lower. The segmental differences in glucose absorption were driven by differences in segmental lengths, as the colon absorbed glucose at a rate which was numerically lower but not significantly different from those of the USI and LSI per cm tissue. The specific focus of this study was to investigate the relationship between the absolute glucose absorption capacity and the resulting secretion of GLP‐1, NT, and PYY secretion in the different intestinal segments. Therefore, a high glucose concentration was administered (20% w/v ~1.1 mol/L). Although not investigated in classical concentration–response manner, the glucose absorption is likely operating at full capacity in our experiments as only 10–20% of the administered glucose was absorbed in the USI and LSI (delivery = 550 *μ*mol/min, absorption = 60–130 *μ*mol/min), whereas in the colon only 6–12% of the infused glucose was absorbed (delivery = 165 *μ*mol/min, absorption = 10–20 *μ*mol/min). The expression of SGLT1 did not fully parallel glucose absorption capacity in the different segmental areas, as higher expression was evident in USI than in LSI, whereas glucose absorption was similar. While we have not been able to narrow down the reason for this discrepancy, it could reflect that the relationship between SGLT1 mRNA and functional protein is complex and/or that SGLT1 activity is regulated posttranslationally. In line with the latter, SGLT1 activity in Xenopus laevis oocytes largely depended on PKC‐ or PKA‐mediated integration of SGLT1 into the membrane (Hirsch et al. 1996; Wright et al. 1997). Additionally, expression analysis on L‐cell marker positive and L‐cell marker negative cells (presumably dominated by enterocytes) from the mouse have shown that GLUT2 expression is substantially lower in both L‐cell positive and L‐cell negative cells from the lower third of the small intestine and colon, compared to the upper third of the small intestine (about 8‐ to 128‐fold lower, respectively) (Reimann et al. 2008). As glucose absorption depends on both transport across the luminal side (presumably mostly mediated by SGLT1 activity but possibly also facilitated by GLUT2 translocation from the basolateral to the apical side of the enterocyte (Kellett et al. 2008)) and exit across the basolateral membranes of the enterocytes (through GLUT2), the low glucose absorption and glucose‐stimulated GLP‐1 secretion in the colon may be related to low levels of GLUT2 expression here compared to the upper small intestine (in both L cells and enterocytes), with basolateral exit of glucose being a rate‐limiting factor for glucose absorption. Indeed, glucose absorption was reduced with about 30% when SGLT1 and GLUT2 were inhibited by luminal administration of the transporter inhibitors phloridzin and phloretin (the latter was added to inhibit potentially translocated GLUT2 transporters). It is noteworthy that the inhibitors only reduced but did not eliminate absorption, and the underlying reason for this clearly requires further investigation. Our observation could, however, be related to that the glucose concentration in these experiments (100 mmol/L) was relatively high compared to other studies of this (Helliwell and Kellett 2002; Mace et al. 2012) albeit not supraphysiological (Ferraris et al. 1990). Supporting this notion, it is generally accepted that glucose absorption comprises two components; a secondary active as well as a facilitated component both of which operate at low glucose concentrations and saturate at 30–50 mmol/L, and a passive component allowing linear increases in absorption up to at least 100 mmol/L (Fullerton and Parsons 1956; Manome and Kuriaki 1961; Lostao et al. 1991; Kellett et al. 2008). The relative roles of the secondary active (SGLT1‐mediated) and the facilitated (GLUT2‐mediated) component is a matter of debate. It is, however, noteworthy that elimination of SGLT1 activity by either genetic knock out (in mice), by pharmacological inhibition or by depletion of sodium ions (which drives SGLT1‐transport from the glucose solution) reduces but does not eliminate glucose absorption in mice (Gorboulev et al. 2012; Röder et al. 2014), isolated perfused rat small intestine loops (Mace et al. 2012) and in humans (Olsen and Ingelfinger 1968). NT secretion showed a similar pattern with fivefold higher rates of secretion from the USI and LSI compared to basal levels. In contrast, NT was undetectable in effluent from the colon (assay detection limit ~1 pM). PYY was predominantly secreted from the LSI where glucose increased the secretion four to fivefold. Glucose also increased the secretion from the USI and colon, but the secretory rate was in both cases 10‐ to 15‐fold lower than that from the LSI (Fig. 4A–G). The regional difference of in particular glucose‐stimulated NT and PYY secretion we find in this study supports the conclusion of two recent papers showing, based on expression analysis of isolated purified murine L cells, that L cells from the upper small intestine differs markedly compared to L cells from the distal small intestine and colon (Egerod et al. 2012; Habib et al. 2012). Indeed, L cells from the upper small intestine resembled CCK producing I‐cells and GIP producing K‐cells more than distal small intestinal L cells, suggesting that the former may comprise a certain rather promiscuous cell type, whereas L cells from the colon are more differentiated and only express GLP‐1 and PYY in appreciable amounts (Habib et al. 2012). Furthermore immunohistochemical studies on mice, rats, and pigs have suggested that the degree of colocalization between GLP‐1 and other intestinal hormones (CCK, GIP, NT, and PYY) differs down the intestine (Cho et al. 2015; Grunddal et al. 2016; Svendsen and Holst 2016), further supporting that the traditional definition of an L cell as a cell type that exclusively expresses GLP‐1 (and the other intestinal proglucagon‐derived peptides glicentin, oxyntomodulin, and GLP‐2) needs revision. Our findings suggest that GLP‐1 secretion is indeed tightly coupled to glucose absorption in the small intestine. As SGLT1 also was expressed in the high levels in the distal part of the colon, it is possible that a similar mechanism is operating here, but probably plays minor role for the total intestinal glucose absorption because (1) the majority of glucose is absorbed in the small intestine; (2) because of the smaller length of the colon; and (3) perhaps because of a relatively lower expression of GLUT2. In fact, very little is known about colonic secretion of GLP‐1, which does not seem to contribute to the GLP‐1 response to meal ingestion, as evidenced by studies in people after total colectomy (Nauck et al. 1996). It is often assumed that colonic GLP‐1 secretion is stimulated by volatile fatty acids generated by fermentation (Psichas et al. 2005; Zhou et al. 2008; Freeland and Wolever 2010; Tolhurst et al. 2012), but this view is challenged by that germ‐free mice (which lack microbial short‐chain fatty‐acid production) have increased rather than decreased basal GLP‐1 plasma levels (Wichmann et al. 2013). Similarly, basal‐ and meal‐induced secretion of GLP‐1 was unaffected in humans after an aggressive antibiotic treatment that resulted in near eradication of gut bacteria (Mikkelsen et al. 2015). Furthermore, in clinical studies there was no relationship between colonic fermentation and GLP‐1 secretion (Ropert et al. 1996). Although the secretory mechanisms for NT appear to be related to those for GLP‐1 (Kuhre et al. 2014b, 2015), the correlation with glucose absorption was much less tight, which is consistent with the finding that the majority of NT‐positive cells are not GLP‐1 positive (Grunddal et al. 2016). Secretion of PYY from ileal cells behaves very much like that of GLP‐1, consistent with their colocalization in the ileal L cells, but is clearly very different in the colon and the USI. On the whole, the many GLP‐1 and PYY secreting cells in the colon are probably poorly related to those in the LSI, which again differ remarkably from those of the USI (neither of which produce or secrete PYY) (Habib et al. 2012). To the extent that the hormones reach the circulation to act on distant target receptors, the importance of their regional origin in relation to the actual response may, however, be less obvious; but it is likely that all three hormones may, at least partly, act by activating local receptors which in turn signals via sensory neurons to, for example, the brain stem and hypothalamus, and in this case the local expression of receptors becomes a question of great relevance.

The finding that gastric bypass‐operated individuals both have accelerated glucose absorption and exaggerated glucose‐stimulated GLP‐1, NT, and PYY secretion (Jacobsen et al. 2013) suggested a link between the two, but this relationship has not been examined previously in detail across the different parts of the intestine. Our results are compatible with the view that under normal circumstances the bulk of glucose is probably absorbed in the USI, whereas the LSI probably serves as a reserve capacity for additional absorption in special cases. Gastric bypass operations for obesity are associated with markedly exaggerated postprandial secretion of several appetite‐ and metabolism‐regulating hormones (increasing up to 30‐fold (Jørgensen et al. 2012)). The underlying mechanisms of this remain to be elucidated, but one hypothesis holds that the diversion of glucose to more distal sites of the intestine could play a key‐role, because the L‐cell density is thought to be greater there (Eissele et al. 1992; Mortensen et al. 2003). As discussed above, this is probably not the case. However, gastric bypass results in an increased rate of nutrient delivery to the distal small intestine, and this undoubtedly explains the increased secretion of PYY. For GLP‐1 the increased secretion is likely to be related to increased glucose absorption (Jacobsen et al. 2013) as supported by the present findings. The rapid absorption rate is likely to be a consequence of the dramatically increased exposure of the small intestine still continuity to glucose‐containing nutrients due to the lack of gastric retention after the gastric bypass operation (Falken et al. 2011).

## Conflict of Interest

The authors of this work declare no potential conflicts of interest relevant to this article.
